# Multimodal representation learning for tourism recommendation with two-tower architecture

**DOI:** 10.1371/journal.pone.0299370

**Published:** 2024-02-23

**Authors:** Yuhang Cui, Shengbin Liang, YuYing Zhang

**Affiliations:** 1 School of Software, Henan University, Kaifeng, China; 2 Institute for Data Engineering and Science, University of Saint Joseph, Macau, China; Sunway University, MALAYSIA

## Abstract

Personalized recommendation plays an important role in many online service fields. In the field of tourism recommendation, tourist attractions contain rich context and content information. These implicit features include not only text, but also images and videos. In order to make better use of these features, researchers usually introduce richer feature information or more efficient feature representation methods, but the unrestricted introduction of a large amount of feature information will undoubtedly reduce the performance of the recommendation system. We propose a novel heterogeneous multimodal representation learning method for tourism recommendation. The proposed model is based on two-tower architecture, in which the item tower handles multimodal latent features: Bidirectional Long Short-Term Memory (Bi-LSTM) is used to extract the text features of items, and an External Attention Transformer (EANet) is used to extract image features of items, and connect these feature vectors with item IDs to enrich the feature representation of items. In order to increase the expressiveness of the model, we introduce a deep fully connected stack layer to fuse multimodal feature vectors and capture the hidden relationship between them. The model is tested on the three different datasets, our model is better than the baseline models in NDCG and precision.

## 1 Introduction

With the rapid development of the Internet and mobile devices, our daily activities connect deeply to online services, such as online shopping, online music and videos. Online services also lead to a surge in data volumes, with information overload making it harder to choose from multitude of services. Recommendation systems (RS) are a powerful information filter for guiding people to find the items of interest in a gigantic and rapidly expanding pool of candidates. Providing users with efficient and accurate prediction results is the goal of RSs. The core methods of RSs include collaborative filtering (CF) [1], content-based recommendation [2] and hybrid recommendation [3]. The principle of the RSs is based on the idea that things are clustered together and people are divided into groups, then recommend items that meet their preferences for users. However, two major challenges faced by existing RSs are the problems of data sparsity and cold start. Data sparsity means that most users interact with only a few items, similarly, many items interact with only a few users. Cold start refers to a new user or item with none interaction, it is difficult for RSs to identify similar candidates and recommend them. The challenging aspect of recommending travel experiences arises from the complexity of multimodal features. In contrast to traditional unimodal data, tourism recommendation tasks require the consideration of information from various perceptual channels, such as images, text, audio, etc., to comprehensively understand users’ needs and preferences. The fusion of this multimodal data introduces a series of technical challenges. In order to solve these problems, the core idea is to add auxiliary features of users and items to build the relationship between users and items.

Recently, the two-tower recommendation model [4–6] is famous for its high efficiency and is widely used in the industry. The notable feature of the two-tower recommendation model is that user and item are two independent sub-networks, one side is the user tower, another side is the item tower, and the parameters of these two towers are not shared. The user tower side covers the features of users, such as user ID, age, historical behavior sequence and so on; the item tower side includes the features of the items, such as item ID, item category, item ratings and so on. The basic workflow of the two-tower model is as follows:

Step 1. the user features and item features are respectively input into the feature extraction network to obtain user embedding and item embedding.
Step 2. calculate the distance between user embedding and item embedding, the items which the user choose are closer, the items which the user do not choose are farther.
Step 3. use the loss function to update the parameters of the model.
Step 4. get all the items embedding through the item tower and store them, then get the top-N candidates for the target user.

Due to the two-tower model suffers from the lack of information interaction between user tower and item tower, and uneven distribution of features in the two towers. Yu et al. [7] proposed a Dual Augmented Two-tower model (DAT), which integrated adaptive mimic mechanism and a category alignment loss. The DAT model effectively alleviates the problems of insufficient information interaction and low query efficiency. A large-scale corpus makes query inefficient. Yang et al. [5] proposed a method called Mixed Negative Sampling (MNS) which uses a mixture of batch and uniformly sampled negatives to address the selection bias of implicit user feedback, the model helps to improve the query speed of features in the recommendation process. Wang et al. [8] applied the two-tower model to the field of video recommendation and explored the possibility of video recommendation based on the two-tower model using different types of metadata. Shen et al. [9] proposed an Adversarial Two-tower Neural Network (ATNN) model for new arrivals CTR predictions. Xu et al. [10] establish some asymptotic results of the two-tower model in terms of its strong convergence to the optimal recommendation system, showing that it achieves a fast convergence rate depending on the intrinsic dimensions of inputs features.

By building an explicit representation on the user-item interactions, literature [11–13] use context information to enrich the representation of users or items, so as to alleviate the cold start problem. Literature [14–16] introduce graph neural network (GNN) into the recommendation model, which can iteratively propagate information from the interactive items, and update the user vector (similarly to item), and can enhance the user/item representation. In order to better incorporate information from heterogeneous interaction types, some solutions such as [17–21] have been proposed in recommendation systems. MBGCN [17] addresses the challenges of data sparsity and cold start issues in traditional recommendation models by constructing a unified graph structure representing various user-item behavioral interactions. It achieves this by learning behavioral intensity and capturing behavioral semantics. HG-GNN [18] is a heterogeneous global graph neural network model that leverages user-item interaction information to better infer user preferences from both current and historical sessions. SCHGN [19] is a self-supervised heat-aware heterogeneous graph network, enhancing food recommendations by considering relationships among ingredients and user preferences for calories. MAINT [20] employs multiple projection mechanisms to capture diverse user preferences and intentions. It utilizes behavior-enhanced LSTM and multi-faceted refined attention mechanisms to adaptively integrate user preferences and intentions, effectively addressing constraints in multi-behavior sequence recommendations. DMRL [21] is a novel recommendation approach that employs disentangled multimodal representation learning, capturing users’ attention to different modalities for each factor in user preference modeling, and outperforms existing methods in extensive evaluations on real-world datasets.

In the field of tourism recommendation, there are various data types for users to evaluate and describe tourism attractions, including traditional manners such as ratings, comments, as well as travel notes, images and short videos. Rational use of these data as features can not only alleviate the cold start problem, but also it is beneficial to improve the quality of recommendation results. Inspired by the above research methods, we propose a multimodal heterogeneous representation learning and apply it to the two-tower recommendation model. In summary, our contributions are:

**A multimodal two-tower recommendation framework**. We showcase how to apply heterogeneous data integrated into the framework to improve recommendation quality. In the item tower, according to different modal information describing items, we use Bi-LSTM [22] model to extract text features from text data, and EANet [23] model to extract image features from images and short videos, then combine them with item ID.

**Heterogeneous representation learning**. In the item tower, there is a large amount of heterogeneous information, and we adopt different strategies to extract features for multimodal information. We designed a deep fully connected stack layer to integrated the features of user tower and item tower, so that the feature latent vectors of each modality extracted are fully cross-fused to capture more deep relationship between user tower and item tower.

**Offline and online experiments**. We conduct extensive offline and online experiments on datasets of different industries and scales, such as tourism, catering and movies to demonstrate the effectiveness of our model. We select a variety of metrics for evaluation, and the experimental results show that our model has better performance. Due to the lack of interaction between the two towers in the early stage, online feature extraction at the user tower is very important. In addition to basic user information, this paper uses Hard Negative Mining (HNM) strategy for negative sampling to extract user features in the online stage, in order to obtain a better user profile.

The rest of this paper is structured as follows. Section 2 introduces the related work. Section 3 introduces the method and framework of our model. Section 4 conducts a variety of numerical experiments and ablation tests to demonstrate the advantage of the model. A brief summary is provided in Section 5.

## 2 Related work

### 2.1 Two-tower model

In the past decade, deep learning has achieved great success in recommendation systems, from using CNN, RNN, GNN to deep reinforcement learning [3]. The two-tower model has two independent sub-networks: user tower and item tower. The core idea is to cache the features of the two towers respectively, an early popular design of two-tower models is DSSM [24]. When online recommendation is performed, it only needs to perform similarity calculation in the memory, which has the characteristics of fast speed and high efficiency. ATNN [9] model predicts the CTR predictions of new arrival by introducing an adversarial network to a two-tower network. The classic DNN [25] model proposed by YouTube is the pioneering work of the two-tower model, and it also includes the recall and ranking. DSSM [24] is a two-tower model for computing semantic similarity, which is used to rank Web documents. Yi et al. [4]. proposed a two-tower model framework for building large scale content-aware retrieval models for industrial scale applications. MIM [26] is a two-tower deep learning model that implements free-form textual descriptive medication phrases inference into patient-friendly medication names. Qin et al. [27] introduced split learning into the two-tower recommendation models and proposed a model called STTFedRec, a privacy-preserving and efficient cross-device federated recommendation framework. MNS [5] used a mixture of batch and uniformly sampled negatives to tackle the selection bias of implicit user feedback. COMET [28] simultaneously utilizes high-order interaction information between historical interactions and embedding dimensions. It captures interaction signals from implicit feedback in the recommendation system and independently learns the representation of the target user and target item.

In summary, the item side of the two-tower model does not depend on the user side, and the massive item-side information uses offline training to improve the efficiency of the model. Existing methods mostly focus on how to improve the feature representation of item-side information, such as positive and negative sampling, to better describe the items. However, this requires a large amount of item information as support, and its effect is limited for small-scale datasets.

### 2.2 Heterogeneous information representation

Although collaborative filtering recommendation systems based on user ratings have better performance, these traditional methods are limited to pre-selected information sources or domain knowledge such as classification standards, descriptions, sentiment analysis and so on, but the recommendation results of such recommendation algorithms lack personalization and they are not universal for some specific application fields. Therefore, researchers develop different recommendation models for user-item interaction in different fields. The recent promising progress in representation learning [29, 30] clarify this problem. With well-established representation learning theories and graph theory on texts [31], images [32], audios [30], videos [33] and many other aspects, we can conduct joint representation learning on heterogeneous information sources in the shared space to obtain more intelligent recommendations. It has an important effect on the rough ranking stage of the recommendation process. Furthermore, by incorporating time sequence relationship [34], context information [35], sentiment analysis [36] and knowledge graph [37] into the ranking stage of recommendation results, we can further optimize the recommendation order. IRGPR [38] model is an innovative re-ranking approach for large-scale commercial recommender systems, utilizing a heterogeneous graph and graph neural network to explicitly model transitive item relationships and personalized user intents, thereby improving the accuracy of re-ranking results compared to existing strategies. RAISE [39] is an advanced re-ranking model that achieves user-specific predictions based on individual intentions, demonstrating superior performance with significant improvements in Precision, MAP, and NDCG on four public datasets. By building representation learning on top of pair-wise learning to rank techniques, we can achieve highly promoted top-N recommendation quality, which is closely related to the business value in the real-world recommendation systems.

In terms of multimodal heterogeneous information representation, the above research has achieved a lot of results. This paper focuses on the efficient representation of implicit features of heterogeneous information such as text, images, and ratings in the item tower, so as to describe a richer description of the items.

## 3 Methodology

In this section, we first give the mathematical definition of a recommendation system based on two-tower architecture, and then construct a feature selection strategy to select candidate items from a high-dimensional, huge corpus; next, we introduce the similarity calculation method between item tower and user tower, and finally, introduce how to train the model.

### 3.1 The architecture of two-tower recommendation model

**Fig 1:**
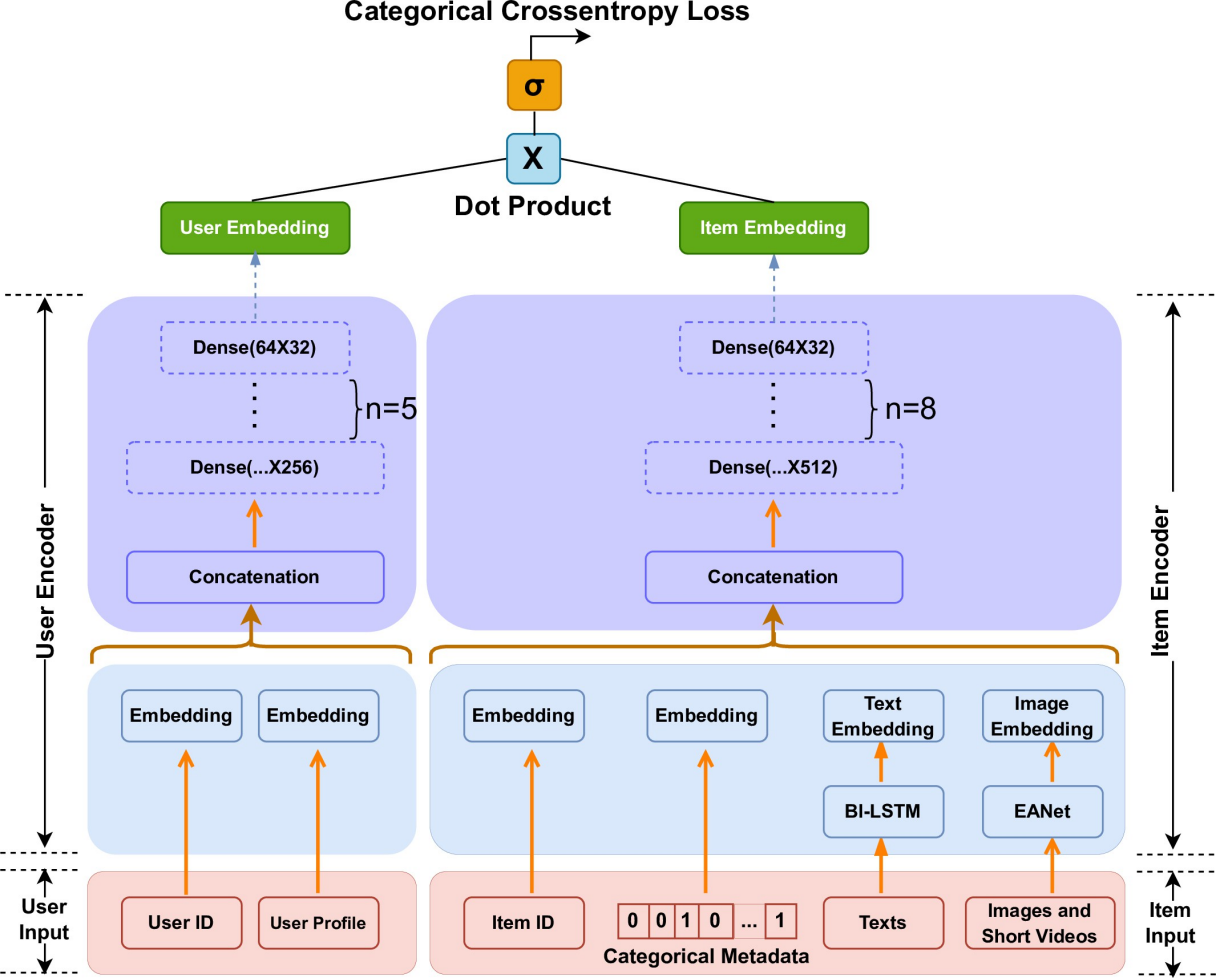
The network architecture of our proposed two-tower DNN model.

The retrieval task in recommendation systems aims to quickly select hundreds to thousands of candidate items from the entire item corpus given a certain query. Given a typical recommendation system with user covariates $x_{u}\in R^{D_{u}}$ and item covariates ${\tilde{x}}_{i}\in R^{D_{i}}$, the two-tower model can be written as

$$R\left(x_{u},{\tilde{x}}_{l}\right)=<f\left(x_{u}\right),\tilde{f}\left({\tilde{x}}_{l}\right)>$$ (1)

where ***f***: *$R^{D_{u}}\to R^{p}$* and $\tilde{f}:R^{D_{i}}\to R^{p}$ are two deep neural networks mapping *x*_*u*_ and ${\tilde{x}}_{l}$ into the same *p*-dementional embedded space. The recommendation mechanism of the two-tower model is based on the dot product of *f*(*x*_*u*_) and $\tilde{f}\left(\tilde{x_{l}}\right)$. Finally, we use the sigmoid function to output the result, as illustrated in Fig 1.

In the tourism recommendation system, the number of features of item tower is much larger than that of user tower. At the same time, the data structure of item tower is more diverse. Many tourism attractions contain a large number of user comments, posted images and short videos. The information basically expresses tourists’ subjective impression of the tourism attractions.

#### 3.1.1 Item tower

The data on the item tower side is multimodal, large in quantity, and extremely complex in structure. We use a variety of neural networks to deal with heterogeneous information, such as text, image and video and structured data.

**Fig 2:**
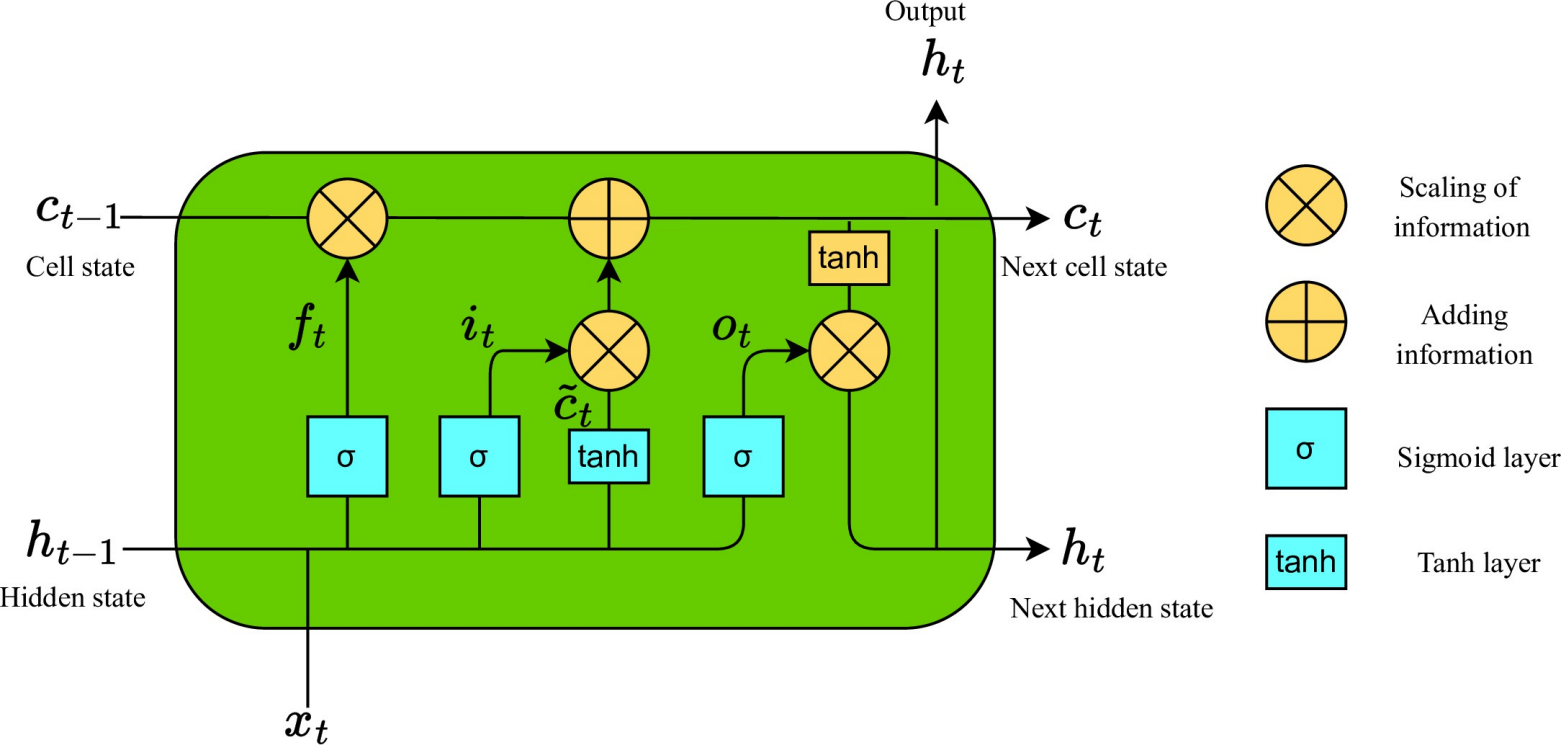
The memory cell structure of LSTM.

*3*.*1*.*1*.*1 Text feature*. LSTM as a deep learning model capable of capturing and understanding sequential data, has demonstrated outstanding performance in recommendation systems. Due to its effectiveness in handling users’ time-series behavioral data, such as click history and purchase records, LSTM excels in capturing both long-term and short-term changes in user interests [40]. By learning temporal correlations and sequence patterns, LSTM can more accurately predict users’ future behavior, thereby enhancing the precision and personalization levels of recommendation systems [3]. Additionally, applying LSTM to user sentiment analysis allows the extraction of user preferences from their reviews, further enriching user features [41]. The traditional recurrent neural network model cannot capture long-distance semantic connection, even if it can transfer semantic information between words. In the process of training, the gradient decreases gradually until it disappears. As a result, the length of sequential data is limited. LSTM overcomes the problem of gradient disappearance by introducing input gate, output gate, forget gate and memory cell. The LSTM network structure is shown in Fig 2.

A LSTM memory cell includes three gates: input gate *i*_*t*_, forget gate *f*_*t*_ and output gate *o*_*t*_. While *c*_*t*_ is cell state representing long-term memory, memory *h*_*t*_ representing short-term memory, and candidate state ${\tilde{c}}_{t}$ waiting to be stored in long-term memory. At time step t, the input gate and the tanh function are used to control the input of new text information at this time, and its value is:

$$i_{t}=\sigma \left(W_{i}*\left[h_{t-1},x_{t}\right]+b_{i}\right)$$ (2)

where *W*_*i*_ is the weight matrix of the input gate, *b*_*i*_ is the bias term of the input gate, *x*_*t*_ is the input feature at the current time step and *σ* is the sigmoid activation function.

The forget gate is the key component of LSTM, which controls the retention and forgetting of memory in the cellular state, and thus avoid the problem of gradient disappearance caused by gradient over time. It is a function of the short-term memory *h*_*t*-1_ of the previous moment and the input *x*_*t*_ of the current moment. And the forget gate is multiplied by the cell state *c*_*t*-1_ of the previous moment to indicate the memory retained from the previous moment. It combines the input gate *i*_*t*_ and the candidate state ${\tilde{c}}_{t}$ to update the cell state to *c*_*t*_, the formula is as follows:

$$\begin{array}{c} f_{t}=\sigma \left(W_{f}*\left[h_{t-1},x_{t}\right]+b_{f}\right),\\ {\tilde{c}}_{\text{t}}=tanh\left(W_{c}*\left[h_{t-1},x_{t}\right]+b_{c}\right),\\ c_{t}=f_{t}*c_{t-1}+i_{t}*{\tilde{c}}_{t}. \end{array}$$ (3)

The output gate *o*_*t*_ is used to control the final output of the entire LSTM, which is combined with the cell state *c*_*t*_ at time *t* to generate the output result of the memory *h*_*t*_ at the current time:

$$\begin{array}{c} o_{t}=\sigma \left(W_{o}*\left[h_{t-1},x_{t}\right]+b_{o}\right),\\ h_{t}=o_{t}*tanh\left(c_{t}\right). \end{array}$$ (4)

Bi-LSTM can solve the problem that LSTM cannot calculate reverse sequence context information, and can combine forward sequence and reverse sequence to output as Eq 5.


$$r_{t}=\vec{h_{t}}∥\overset{\leftarrow }{h_{t}}$$ (5)

Among them, $\vec{h_{t}}$ and $\overset{\leftarrow }{h_{t}}$ are the results of the forward and reverse hidden layers of Bi-LSTM respectively, Bi-LSTM is calculated and updated in two directions, both sequences are directly connected to the output layer, providing complete contextual state for each word.

**Fig 3:**
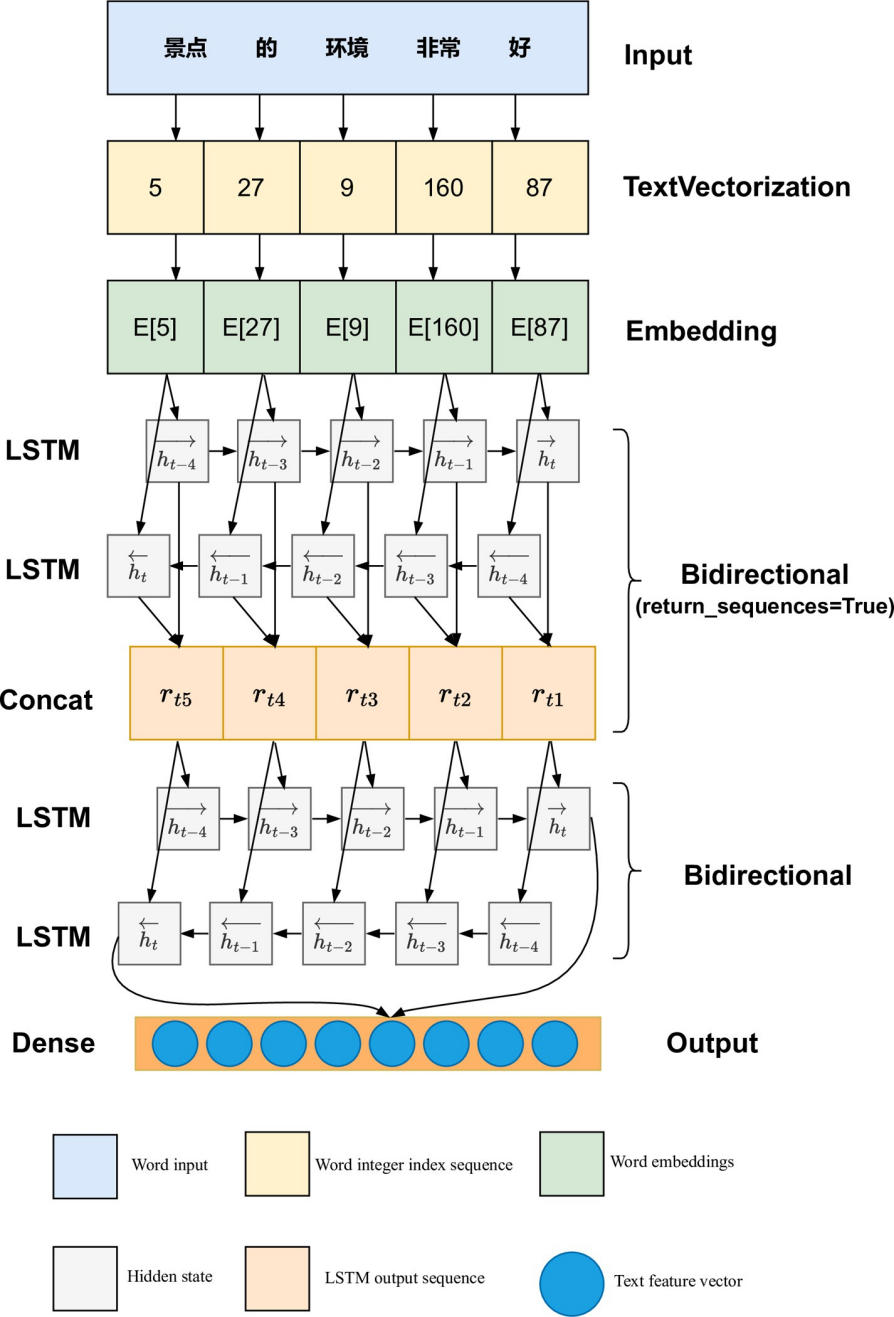
The workflow of text feature processing.

In the recommendation model, we design a two-layer Bi-LSTM for processing comments, in the first layer of Bi-LSTM, we output *h*_*t*_ at each time step (return_sequences = True), and connect it as the input of the second Bi-LSTM layer. For example, the text such as “景点的环境非常好” (The environment of the attraction is very good). Firstly, the Chinese comments are segmented, then these words are converted into word embedding. Secondly, these word embeddings are sequentially input into the Bi-LSTM model to obtain the output vector *r*_*t*_. Finally, we send *r*_*t*_ to a fully connected layer with 32 neurons and convert it into a one-dimensional vector of length 32, which fits the text features required for the recommendation model. The process is shown in Fig 3.

*3*.*1*.*1*.*2 Image and short video feature*. We use EANet to extract features from images and videos. Since self-attention ignores the potential correlation between different samples, EANet proposes a new external attention mechanism to replace self-attention, which is based on two external, small-scale, learnable, shared memory. While external attention implicitly considers the correlations between samples, which helps us to mine the potential preferences of users. EANet further incorporates multi-head attention into the external attention mechanism, and has achieved very good results in tasks such as computer vision. We design an external attention MLP framework for extracting image features describing tourism attractions, and integrates it into our two-tower recommendation model.

**Fig 4:**
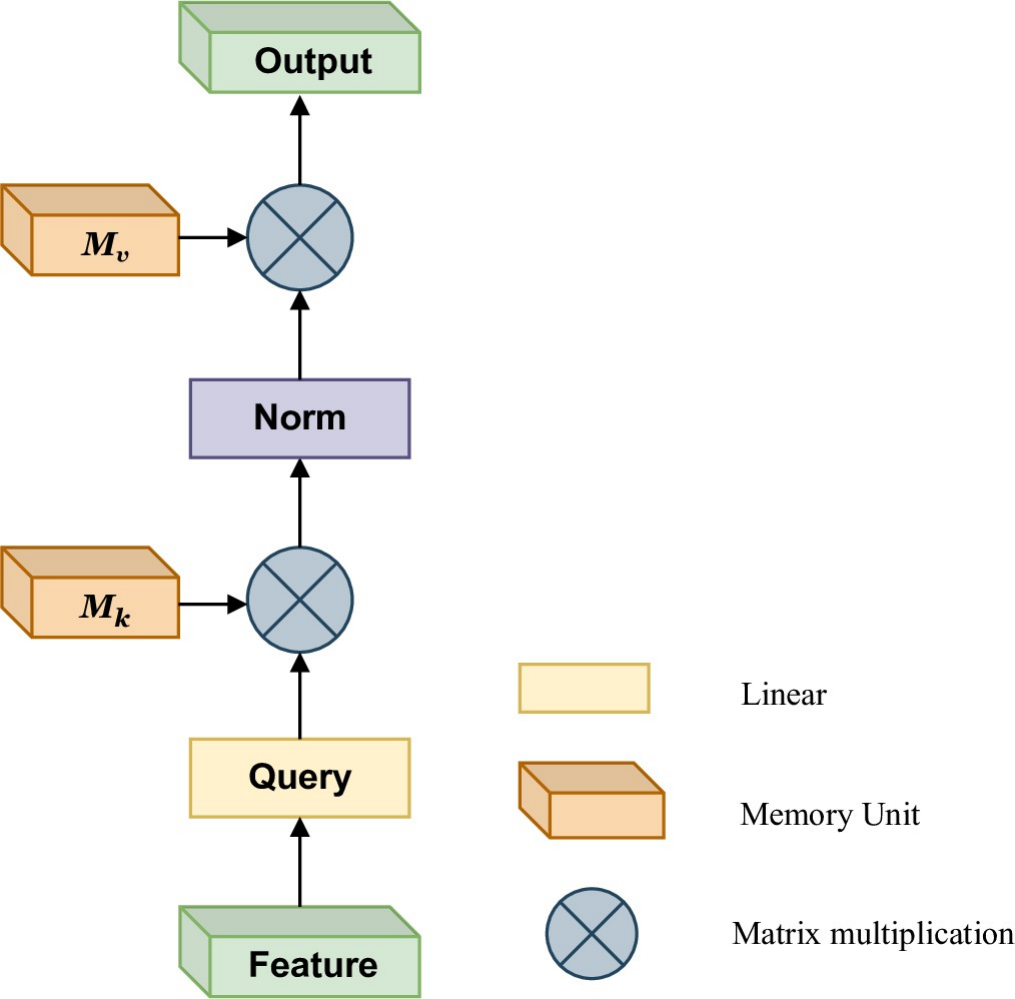
External-attention.

The external attention mechanism is shown as Fig 4, We first calculate the attention map by computing the affinities between the self-query vectors and an external learnable key memory, and then produce a refined feature map by multiplying this attention map by another external learnable value memory. They are independent of individual samples and shared across the entire dataset, which plays a strong regularization role and improves the generalization capability of the attention mechanism. The external memories are designed to learn the most discriminative features across the whole dataset, capturing the most informative parts, as well as excluding interfering information from other samples. The structure of external attention mechanism is simple, but it is very effective for various computer vision tasks. Because of its simplicity, the TTHM model proposed in this paper integrates it to obtain the features contained in the images’ description of tourism attractions.

External attention utilizes Eq 6 to compute the attention map between the input pixels and an external memory unit $M\in {\mathbb{R}}^{S\times d}$,

$$\begin{array}{c} A={\left(\alpha \right)}_{i,j}=\text{Norm}\left(FM^{T}\right),\\ F_{out}=AM. \end{array}$$ (6)

Among them, (*a*)_*i*,*j*_ is the similarity between the *i*th pixel and the *j*th row of *M*, where *M* is a learnable parameter independent of the input, which acts as a memory of the whole training dataset. *A* is the attention map inferred from this learned dataset-level prior knowledge. Finally, we update the input features from *M* by the similarities in *A*. To enhance the network’s capability, we use two different memory units *M*_*k*_ and *M*_*v*_, as the key and value.

The attention map is sensitive to the scale of the input features. External attention does not use the softmax function to normalize the attention map but utilizes double normalization [42] to normalize the columns and rows. The double normalization is described as Eq 7,

$$\begin{array}{c} {\left(\tilde{\alpha }\right)}_{i,j}=FM_{K}^{T},\\ {\hat{\alpha }}_{i,j}=\frac{\exp\left({\tilde{\alpha }}_{i,j}\right)}{\sum_{k}\exp\left({\tilde{\alpha }}_{k,j}\right)},\\ \alpha_{i,j}=\frac{{\hat{\alpha }}_{i,j}}{\sum_{k}{\hat{\alpha }}_{i,k}}. \end{array}$$ (7)

**Fig 5:**
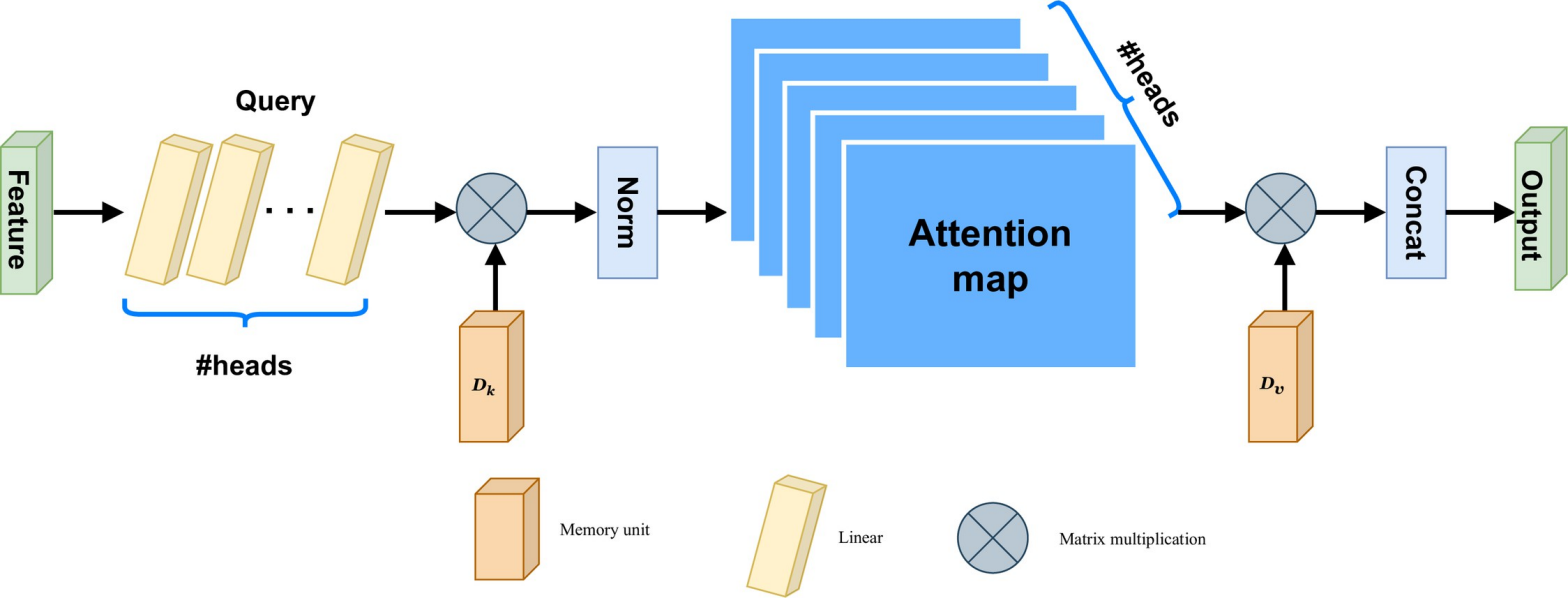
Multi-head external attention.

In Transformer [43], self-attention is computed many times on different input channels, which is called multi-head attention. Multi-head attention can capture different relations between tokens, improving upon the capacity of single head attention, external attention uses a similar method, the structure of external attention is shown as Fig 5.

Multi-head external attention descripted as Eq 8,

$$\begin{array}{c} h_{i}=\text{Extental}\;\text{Attention}\left(F_{i},M_{k},M_{v}\right),\\ F_{out}=\text{MultiHead}\left(F,M_{k},M_{v}\right)=\text{Concat}\left(h_{i},\ldots ,h_{H}\right)W_{O}. \end{array}$$ (8)

where *h*_*i*_ is the *i*th head, *H* is the number of heads and *W*_*O*_ is a linear transformation matrix making the dimensions of input and output consistent. $M_{k}\in {\mathbb{R}}^{S\times d}$ and $M_{v}\in {\mathbb{R}}^{S\times d}$ are the shared memory units for different heads. For image data, the basic flow of its processing is as follows:

Step 1. The image data is converted into a 32*32*3 gray scale image through the input layer;
Step 2. Then gray scale images go through a patch extract layer and a patch embedding layer to extract the key features of the tourism attractions;
Step 3. The initially extracted features are passed through the EANet model described above to obtain detailed image features, which contain the potential correlation between all samples;
Step 4. Output the final result through a fully connected layer with 32 neurons, which is a one-dimensional vector of length 32.

*3*.*1*.*1*.*3 Structured data*. In addition to the above features, other features on the item tower side are structured data, our model use embedding layer convert them into embedded vectors of low dimension with a fixed size. Suppose there are *n* structured features at the item tower side, *K*_*j*_ is the dimension of the embedding layer corresponding to the jth feature. For each feature *f*_*i*_ of ${\tilde{x}}_{l}$, map to a low-dimensional dense layer through the corresponding embedded vector $e_{i}\in {\mathbb{R}}^{K_{i}}$, then all the embedded vectors at the item tower side E∈ℝ^*L*^, where L is the dimension of E, that is, **$\text{L}=\sum_{i=1}^{n}K_{i}$**.

Finally, In the item tower side, we perform the full connection operation on the BI-LSTM and EANet output results, and the embedded vectors generated by the structured data to obtain ${\tilde{x}}_{l}$ in Eq 1.

#### 3.1.2 User tower

The main features of the user tower include user ID and user profile. These features are basically structured data, so use the embedding layer to convert them into fixed dimensional embedded vectors. Then we use the full connection operation to concatenate the embedded vectors to generate user covariates *x*_*u*_ in Eq 1.

#### 3.1.3 Deep fully connected stack layer

We perform the corresponding concatenation operation on all the embedded vectors at both sides of the user tower and item tower, and obtain two fusion vectors *z*_*u*_ and *z*_*i*_. They are calculated as in Eq 9.

$$\begin{array}{l} z_{u}=\left[embedding_{uid}∥embedding_{age}∥...∥embedding_{gender}\right],\\ z_{i}=\left[embedding_{iid}∥embedding_{rating}∥embedding_{price}∥\cdots ∥h_{t}∥y_{i}\right]. \end{array}$$ (9)

where || represents the vector concatenation operation. In this way, the final fusion vector contains various of multimodal features.

**Fig 6:**
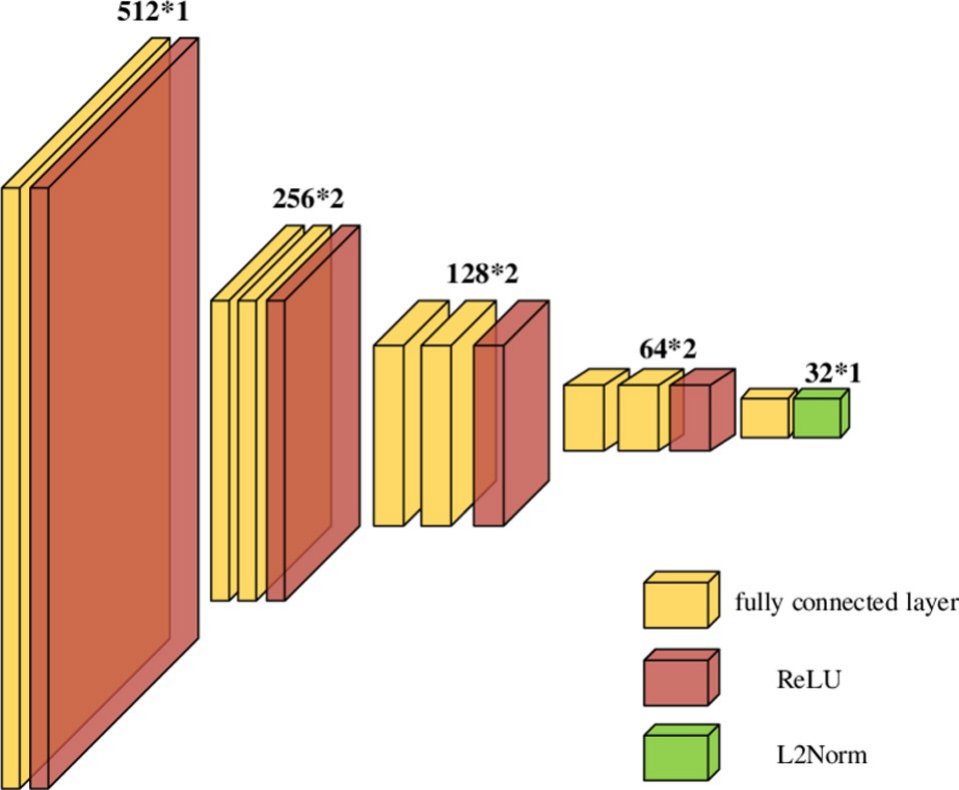
The structure of fully connected layer.

After getting *z*_*u*_ and *z*_*i*_, we feed them into the corresponding two towers, which are stacked by gradually narrowing fully connected layer. We use ReLU activation function for the fully connected layer. Finally, the output result of the fully connected layer will pass through L2 regularization layer to obtain the fusion vectors *p*_*u*_ and *p*_*i*_ with fully crossed features, its structure is shown in Fig 6.

We use Eq 10 to describe the operation of the fully connected layer,

$$\begin{array}{c} {\text{d}}_{\text{j}}=ReLU\left(w_{\text{j}}{\text{z}}_{u,i}+{\text{b}}_{\text{j}}\right),\\ {\text{d}}_{\text{L}}=ReLU\left(w_{l}{\text{d}}_{\text{L}-1}+{\text{b}}_{l}\right),\\ {\text{p}}_{u,i}=L2\cdot \text{Norm}\left({\text{d}}_{\text{L}}\right). \end{array}$$ (10)

Where d_j_ denote the jth fully connected operation, w_j_ and b_j_ are the weight and bias vector of the jth fully connected layer, *z*_*u*,*i*_ represents *z*_*u*_ and *z*_*i*_, p_*u*,*i*_ represents the output vector of the L2 regularization layer.

Finally, our model calculates the user-item similarity using the dot product as shown in Eq 11, where *z*_*i*_ and *z*_*u*_ represent the embedding of the item and user respectively.


$$\text{Similarity}=\cos\theta =\frac{{\text{z}}_{i}\cdot {\text{z}}_{u}}{\left\|z_{i}\|\|z_{u}\right\|}=\frac{\sum_{k=1}^{n}z_{i_{k}}\times z_{u_{k}}}{\sqrt{\sum_{k=1}^{n}{\left(z_{i_{k}}\right)}^{2}}\times \sqrt{\sum_{k=1}^{n}{\left(z_{u_{k}}\right)}^{2}}}$$ (11)

### 3.2 Metadata

In this section, we introduce three types of metadata for the tourism recommendation: category feature, text feature and image feature. Category feature is a subset of metadata that generally listed by online tourism server providers, which are classifications of different types of tourism attractions. We first represent each categorical feature with a one-hot code. The categorical features mainly include the following five types:

- Ticket price is the primary concern of users, since the ticket prices of various tourism attractions are different, if each price is considered separately, the feature coding will be very lengthy. In order to simplify this process, we divide the ticket prices into four levels: free, cheap, medium and expensive.
- The recommended tour duration is the amount of time users need to visit the attractions, and we divide it into ten levels at hourly intervals.
- Opening hours indicate the opening hours of attractions, and we divide them into three levels: short, medium and long.
- Ratings indicate the overall evaluation of the attraction by all tourists, divided into five grades from 1 to 5 points.
- The popularity of attractions represents the number of tourists in a fixed period, we set the popularity to five levels according to the number of tourists.

After we get the one-hot representation of each categorical feature, we concatenate it into a binary vector, which is then converted into a dense vector through an embedding layer.

The text features are mainly commenting on attractions in the online community, and various comments on attractions from the user’s point of view include service level, experience, etc. Since the text lengths of these corpora are different, and most of them are Chinese, we firstly perform word segmentation with Chinese word segmentation tools; then encode each word, in order to avoid the long tail problem, we keep the 20,000 most frequent words. They are converted into a 64-dimensional dense vector by embedding; finally, we use a two-layer Bi-LSTM to extract a 32-dimensional vector from the embedding layer.

Image features are very important way of describing tourist attractions. Images can increase people’s cognition of tourism attractions and show the characteristics of attractions. We extract relevant attractions from the user-item interaction history, and use the EANet model to extract image feature to 32-dimensional vectors. The feature vector that distinguishes different types of attractions from the input images, by comparing from different images of the same type of attractions are very close in the feature space, and different types of attractions are far apart in the feature space. The type of attractions is determined through feature extraction and comparison.

### 3.3 Model training

We introduce the structure of the model and the fusion and representation of multimodal features. In the recall phase of the recommendation system, in most cases, there are usually close semantic associations between candidates, and it is necessary to match the text at a finer attribute granularity. Appropriately increasing the difficulty of strong negative examples in the training data will help improve the model effect. The general practice is to sample from a ranked candidate paragraph, and the higher the negative example, the more difficult it is for the model. However, due to the unavoidable missing labeling situation, direct sampling has a high probability of introducing false negatives. We use the HNM strategy for negative sampling. In the model training stage, in addition to the positive samples, the negative sampling method is used to incorporate the same data that is mistaken as a negative sample into the training set.

Step 1. Using Eq 12, determine the negative sample, that is, the sample whose predicted value differs greatly from the label.
Step 2. Based on the logits, determine the number of negative sampling samples, our model sets the number of negative sampling samples as ***S***.
Step 3. Calculate the total number of samples ***T*** in the training set according to Eq 13.
Step 4. Model calculation, and output recommendation results.

$$loss_{p}=-\frac{1}{T}\sum_{(u,i,y)\in T}\left(y\;\log\;\sigma \left(<p_{u},p_{i}>\right)+(1-y)\log\left(1-\sigma \left(<p_{u},p_{i}>\right)\right)\right)$$ (12)

T=D×(S+1) (13)

where ***D*** is the number of positive feedback query-item pairs, ***T*** is the total number of train pairs, and ***σ*** represents the sigmoid function.

## 4 Experimental results

We perform experiments on one public dataset and one industrial dataset to fully evaluate our model. Furthermore, we conduct extensive ablation test on each proposed component. We also provide a visualization comparison with five other models.

### 4.1 Datasets

To evaluate the effectiveness of our model, we conduct extensive experiments on three different datasets: Tourism, MovieLens and Grocery & Food. The Tourism is a dataset about tourism topics and its data is collected from mafengwo.cn. These datasets are tourism-related datasets, and their data scale and number of interactions are quite different, including both Chinese and English corpora, which can more fully reflect the performance of our model. Table 1 summarizes the statistics of all the datasets.

**Table 1:** Statistics of evaluation datasets.

Dataset	Users	Items	Interactions	Language
Tourism	3531	266	63157	Chinese
MovieLens	610	9743	100837	English
Grocery & Food	774095	120774	1997599	English

### 4.2 Evaluation metrics

For all experiments, we evaluate our model and baseline in terms of Precision@k, Recall@k, F1-value@k and NDCG@k, we report with k = 5 and 10.

- *Precision@k*: The metric represents the accuracy of the recommendation system, that is the proportion of relevant results that were correctly predicted out of all the results returned. The metric is often used to measure the precision of the recommendation system. It is computed as:

$$Precision@k=\frac{TP@k}{TP@k+FP@k}$$ (14)

True positive (TP) stands for true examples, false positive (FP) stands for false positive example, False negative (FN) stands for false counterexample. The value of *Precision @k* range is [0,1], the bigger the better.

- *Recall@k*: The metric represents the proportion of the number of samples predicted to be positive in all positive samples. It is useful for evaluating the performancSe of recommendation systems and can be used to make improvements to the system. By optimizing the system to increase the number of relevant items recommended, the overall user experience can be improved. Additionally, the metric can be used to compare the performance of different recommendation systems and determine which one is more effective. Is can be expressed as:

$$\text{Recall@k}=\frac{TP@k}{TP@k+FN@k}$$ (15)

- *F1@k*: The metric is the harmonic mean of Precison@k and Recall@k. It used to evaluate how many relevant items have been retrieved among the top k results returned by the model. Essentially, it measures the proportion of relevant items that were retrieved in the top k results. It can be expressed as:

F1@k=2×Precision@k×Recall@kPrecision@k+Recall@k (16)

- *NDCG@k*: The Normalized Discounted Cumulative Gain (NDCG) is one the most frequently used evaluation measure, which considers the position of correctly recommended items. NDCG is averaged across all testing users. It is computed as:

$$\text{NDCG}@\text{k}=\frac{DCG_{k}}{I{\text{DCG}}_{k}}=\frac{\sum_{i=1}^{k}\frac{r{\text{el}}_{i}}{\log_{2}(i+1)}}{\sum_{i=1}^{\left|REL\right|}\frac{r{\text{el}}_{i}}{\log_{2}(i+1)}}$$ (17)

where *r*el_*i*_ refers to the true relevance score of the *ith* result. |*REL*| indicates that the results are sorted according to the true correlation from large to small, and the number of sets consisting of the first *k* results is taken.

### 4.3 Baseline algorithms

To demonstrate the effectiveness, we compare and analyze our proposed TTHM model with the following baselines. Among these models, there are recommendation systems based on Bayesian methods, collaborative filtering algorithm based on neural network method, heterogeneous information recommendation models based on two-tower architecture, and recommendation system based on graph neural networks. They are state-of-the-art methods among different recommendation methods.

#### 4.3.1 BPR [44]

Bayesian Personalized Ranking (BPR) is an approach for personal recommendation, it generates a recommendation list based on implicit feedback. It assumes that different behavior reflects different order preference between user and item, and this can be used as prior knowledge to build more diverse training pairs.

#### 4.3.2 NCF [45]

Neural Collaborative Filtering (NCF) is a classical deep neural network model for RS, NCF is a general framework that can model user-item interactions in different ways. NCF complements the mainstream shallow models for collaborative filtering, NCF proposes to leverage a multi-layer perceptron to learn the user–item interaction function.

#### 4.3.3 HERec [46]

HERec is designed to solve the problem of heterogeneous information network, it includes a meta-path based random walk strategy to generate meaningful node sequences for network embedding. The learned node embeddings are first transformed by a set of fusion functions, and subsequently integrated into an extended matrix factorization (MF) model. The extended MF model together with fusion functions are jointly optimized for the rating prediction task.

#### 4.3.4 MoHINRec [47]

MoHINRec is a motif-enhanced meta-path, which further captures the high-order relationship between nodes of the same type, and then inputs the embedding representation into the factorization machine for training.

#### 4.3.5 ATTR [48]

ATTR is a neural sequence recommendation model designed for scenic spots, employing a Self-Attention mechanism to capture sequence representations and model multiple relationships within items. This individualized Tourism Recommendation system analyzes user interactions through self-attention, capturing both long- and short-term preferences. The model enhances item embedding by preserving the relationship structure between scenic items, enabling accurate analysis of user interests for effective item prediction.

#### 4.3.6 TMFUN [49]

TMFUN is a novel and effective Attention-guided Multi-step Fusion Network designed for multimodal recommendation. The main objective of this model is to leverage the rational utilization of item multimodal information for enhanced recommendation performance. In contrast to previous approaches that directly integrate multimodal features with item ID embeddings, TMFUN focuses on preserving inherent semantic relations within multimodal features. The model constructs modality feature graphs and item feature graphs to capture latent item-item semantic structures.

#### 4.3.7 SGFD [50]

SGFD is a model-agnostic approach, Semantic-guided Feature Distillation, devised to address challenges in multimodal recommendation. It employs a teacher-student framework to enhance feature extraction. The teacher model extracts rich modality features by considering semantic information and complementary details from multiple modalities. In this paper, we use SGFD-GRCN as the baseline.

### 4.4 Parameter settings

We optimize all models using the Adam optimizer, and the experimental hyperparameter settings in our model are shown in Table 2.

**Table 2:** Parameter settings.

Experimental parameters	Value
LSTM units	64*4
Num_heads	4
Multiple stacked dense dim (query)	256*1,128*2,64*1,32*1
Multiple stacked dense dim (item)	512*1,256*2,128*2,64*2,32*1
Optimizer	Adam (lr = 1e-5)
Random rate	0.9

### 4.5 Result and discussion

Tables 3–5 report the overall performance compared with baselines. Each result is the average performance from 10 runs with random weight initializations.

**Table 3:** Experimental results on Tourism dataset.

Model	BPR	NCF	HERec	MoHINRec	ATTR	TMFUN	SGFD	TTHM
Precision@5	0.218	0.244	0.259	0.272	0.256	0.281	**0.286**	0.284
Precision@10	0.223	0.316	0.355	0.359	0.367	0.369	**0.375**	0.372
Precision@20	0.225	0.327	0.360	0.355	0.369	0.377	0.383	**0.385**
Precision@50	0.237	0.332	0.367	0.371	0.373	0.380	0.382	**0.388**
Recall@5	0.244	0.264	0.278	0.270	0.266	**0.281**	0.274	0.279
Recall@10	0.282	0.313	0.336	0.341	0.338	0.352	0.355	**0.359**
Recall@20	0.287	0.319	0.342	0.355	0.326	0.363	0.360	**0.367**
Recall@50	0.295	0.323	0.350	0.367	0.346	0.371	0.373	**0.375**
F1@5	0.249	0.253	0.268	0.276	0.270	0.276	**0.281**	0.279
F1@10	0.257	0.314	0.345	0.349	0.342	0.360	0.362	**0.365**
F1@20	0.286	0.342	0.383	0.390	0.379	0.397	0.393	**0.409**
F1@50	0.299	0.357	0.398	0.402	0.385	0.415	0.412	**0.420**
NDCG@5	0.519	0.559	0.611	0.637	0.648	0.689	0.693	**0.696**
NDCG@10	0.541	0.580	0.604	0.611	0.609	0.631	0.628	**0.633**
NDCG@20	0.556	0.572	0.620	0.645	0.641	0.655	0.660	**0.662**
NDCG@50	0.527	0.589	0.647	0.658	0.653	0.674	**0.677**	0.670

**Table 4:** Experimental results on MovieLens dataset.

Model	BPR	NCF	HERec	MoHINRec	ATTR	TMFUN	SGFD	TTHM
Precision@5	0.0145	0.0162	0.0175	0.0196	0.0171	0.0201	0.0204	**0.0207**
Precision@10	0.0334	0.0347	0.0388	0.0391	0.0379	0.0415	0.0413	**0.0418**
Precision@20	0.0355	0.0362	0.0396	0.0417	0.0384	0.0436	0.0433	**0.0439**
Precision@50	0.0379	0.0387	0.0405	0.0424	0.0407	0.0457	**0.0459**	0.0455
Recall@5	0.0125	0.0133	0.0152	0.0173	0.0156	0.0176	0.0180	**0.0184**
Recall@10	0.0230	0.0259	0.0295	0.0309	0.0293	0.0311	0.0313	**0.0316**
Recall@20	0.0261	0.0282	0.0311	0.0325	0.0305	0.0343	0.0347	**0.0351**
Recall@50	0.0276	0.0291	0.0335	0.0346	0.0329	0.0360	0.0365	0.0368
F1@5	0.0134	0.0146	0.0162	0.0183	0.0166	0.0188	0.0192	**0.0194**
F1@10	0.0288	0.0297	0.0330	0.0340	0.0337	0.0357	0.0358	**0.0360**
F1@20	0.0307	0.0329	0.0337	0.0356	0.0345	0.0374	0.0372	**0.0377**
F1@50	0.0325	0.0342	0.0355	0.0370	0.0358	0.0387	0.0384	**0.0392**
NDCG@5	0.082	0.086	0.091	0.102	0.097	**0.117**	0.113	0.115
NDCG@10	0.089	0.095	0.113	0.117	0.105	0.120	**0.127**	0.124
NDCG@20	0.085	0.088	0.092	0.115	0.090	0.124	**0.129**	0.126
NDCG@50	0.092	0.096	0.102	0.119	0.105	0.125	0.126	**0.132**

**Table 5:** Experimental results on Grocery & Food dataset.

Model	BPR	NCF	HERec	MoHINRec	ATTR	TMFUN	SGFD	TTHM
Precision@5	0.0178	0.0204	0.0223	0.0246	0.0229	0.0251	0.0250	**0.0255**
Precision@10	0.0164	0.0219	0.0206	0.0257	0.0237	0.0253	**0.0259**	0.0241
Precision@20	0.0176	0.0227	0.0231	0.0252	0.0240	0.0264	0.0262	**0.0268**
Precision@50	0.0182	0.0238	0.0247	0.0263	0.0245	0.0270	0.0269	**0.0273**
Recall@5	0.0422	0.0467	**0.0519**	0.0484	0.0477	0.0512	0.0515	0.0497
Recall@10	0.0515	0.0532	0.0566	0.0551	0.0560	0.0574	0.0572	**0.0578**
Recall@20	0.0492	0.0547	0.0553	0.0573	0.0558	0.0577	0.0579	**0.0582**
Recall@50	0.0522	0.0531	0.0566	0.0584	0.0542	0.0590	0.0588	**0.0592**
F1@5	0.0250	0.0283	0.0312	0.0326	0.0318	0.0335	0.0331	**0.0337**
F1@10	0.0248	0.0311	0.0302	0.0351	0.0337	**0.0353**	0.0348	0.0340
F1@20	0.0259	0.0320	0.0328	0.0341	0.0345	0.0356	0.0354	**0.0358**
F1@50	0.0262	0.0331	0.0340	0.0352	0.0354	0.0364	**0.0371**	0.0367
NDCG@5	0.096	0.115	0.131	0.147	0.153	0.158	0.160	**0.162**
NDCG@10	0.092	0.107	0.140	0.155	0.157	0.160	0.162	**0.164**
NDCG@20	0.095	0.118	0.148	0.167	0.162	0.169	0.168	**0.173**
NDCG@50	0.102	0.113	0.167	0.156	0.171	0.177	**0.182**	0.179

Table 3 show the experimental results on Tourism dataset. Tourism dataset is a Chinese corpus with relatively few interactions. The experiment results show that our model outperforms all baseline models in terms of most classification indicators such as precision, F1 and ranking indicators NDCG.

Table 4 show the experimental results on MovieLens dataset. The dataset is an English corpus with a greater number of interactions. The experimental results show that our proposed TTHM model outperforms the baseline models in both F1 and Recall. It indicates that TTHM model performs better on medium-scale dataset.

Table 5 show the experimental results on Grocery & Food dataset. Grocery & Food dataset is the biggest among the three datasets. Due to Grocery & Food dataset has a large number of category features compared to the previous two datasets, for example, each food has a large number of ingredient lists. TTHM model focuses on text features and image features, and only uses a simple embedding layer for classification features to convert them into low-dimensional embedding vectors. It makes the model not fully extract and integrate a large number of classification features. It shows that TTHM model has some limitations, that is, the representation of categorical feature embedding with many features needs to be improved.

### 4.6 Ablation studies

We conduct ablation studies on Tourism and MovieLens dataset to justify its effectiveness. As a heterogeneous two-tower recommendation model, TTHM introduces different categories and quantities of data, and its recommendation results are also different. In this ablation tests, firstly, we only added categories as feature to the model and used it as a benchmark. Secondly, we add heterogeneous data such as texts and images to the neural network of the item tower, then fused the features to get the recommendation results respectively. Finally, further modify the model structure and establish a fully connected stack layer between the user tower and the item tower to recommend heterogeneous data. At different stages, the accuracy of different structural models is significantly different. The experimental results in the three datasets are shown in Tables 6–8, Connect denote concatenate all types of metadata directly, DDS means to fuse different types of metadata using fully connected layer.

**Table 6:** Ablation tests results on MovieLens dataset.

Data and Architecture	P@1(%)	P@5(%)	P@10(%)	P@50(%)
Categories	-	-	-	-
Text	97.5	63.8	14.7	28.8
Image	380.7	130.5	56.72	40.8
Connect	482.6	154.2	118	104.3
DDS	531	165	130.9	124.1

**Table 7:** Ablation tests results on Tourism dataset.

Data and Architecture	P@1(%)	P@5(%)	P@10(%)	P@50(%)
Categories	-	-	-	-
Text	447.6	14.7	68.7	14.4
Image	110.19	56.72	11	7.3
Connect	525.7	118	95.6	20.2
DDS	633.1	130.9	78.5	25.3

**Table 8:** Ablation tests results on Grocery & Food dataset.

Data and Architecture	P@1(%)	P@5(%)	P@10(%)	P@50(%)
Categories	-	-	-	-
Text	245.7	208.5	68.7	177.9
Image	330.1	310.7	11	266
Connect	420	394	95.6	342.3
DDS	467.8	422.2	78.5	387.7

**Fig 7:**
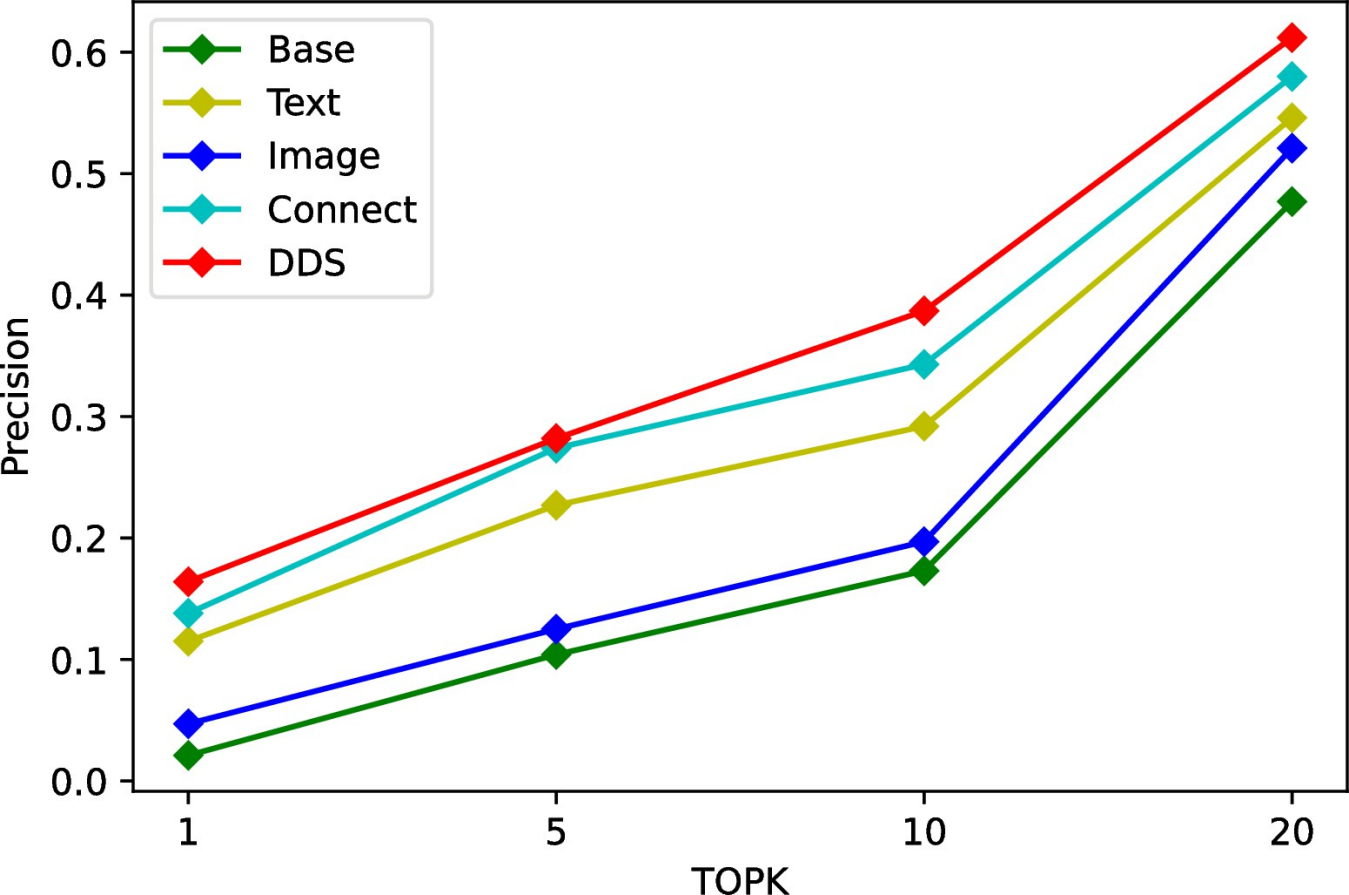
Ablation tests on Tourism dataset.

**Fig 8:**
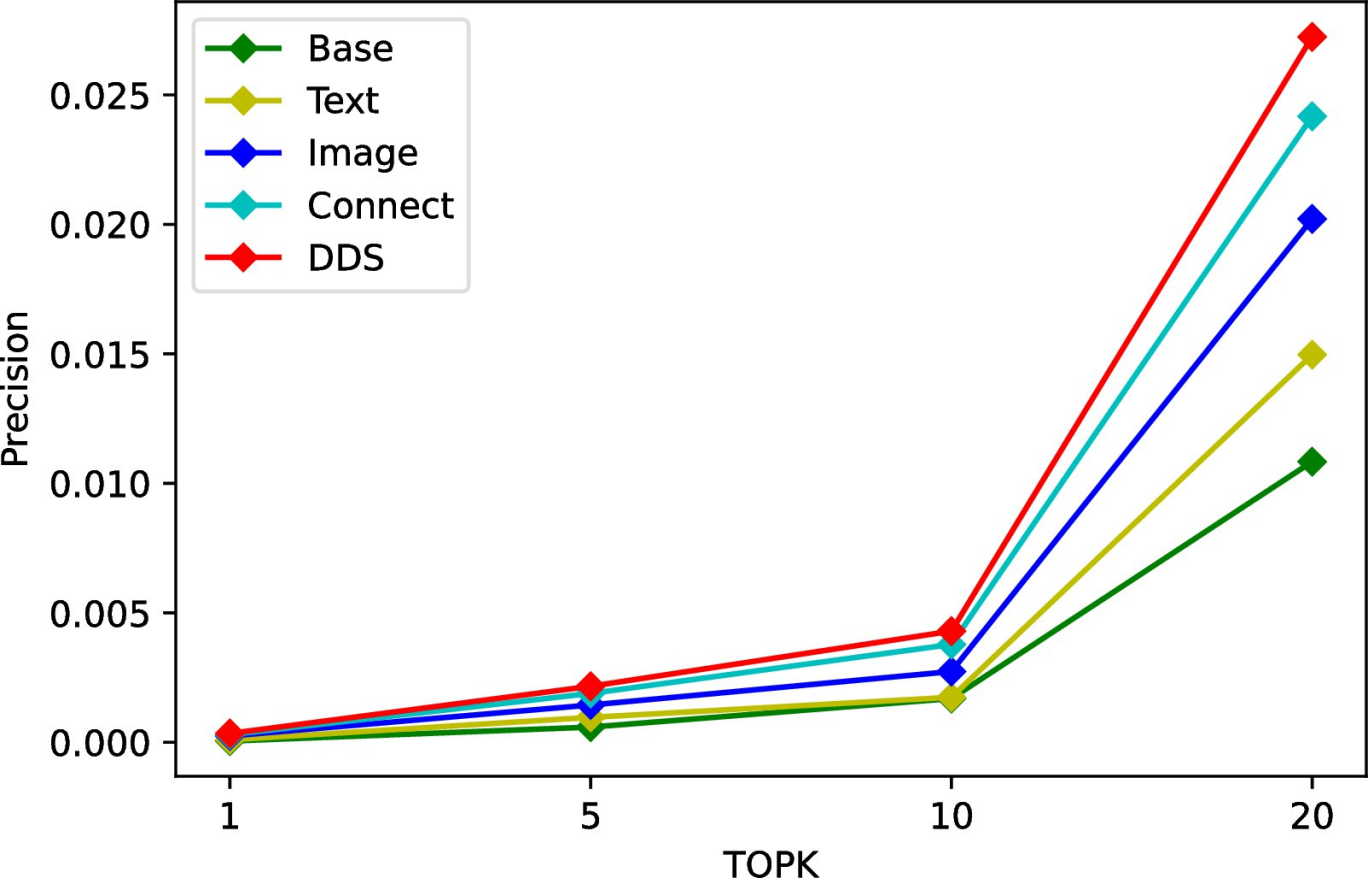
Ablation tests on MovieLens dataset.

**Fig 9:**
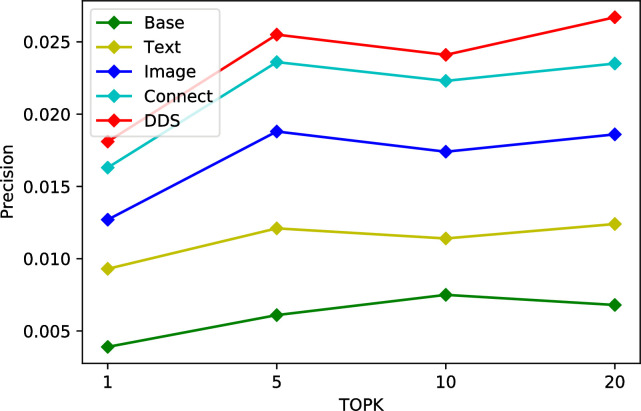
Ablation tests on Grocery & Food dataset.

The experimental results are shown in Figs 7–9. Compared with the basic features, multimodality features are gradually integrated with the introduction of various features, the accuracy of the model continues to improve. But the importance of each feature is different, as we can see, for the Tourism dataset, the text features are significantly better than the image features, but the opposite is true for the MovieLens dataset. Compared to simply concatenating individual features, the fully-connected stack layers can bring significant improvements to the model, suggesting that the introduction of fully-connected stack layers is beneficial.

## 5 Conclusion

In this paper we have presented a novel heterogeneous two-tower recommendation model TTHM with two key components: feature fusion method for heterogeneous data and fully connected stack layer in two-tower framework. We discussed different heterogeneous data such as texts, images, videos into feature fusion. Compared to the baseline models, the overall performance of TTHM is good, especially in the terms of NDCG, which is better than the baseline models in the three datasets, indicating that our model is better in ranking. At the same time, there are certain advantages in Recall, Precision and F1, especially for small and medium-scale datasets. We will solve the problem of classification performance on large-scale dataset in the future.

For future work, we also plan to conduct experiment on online systems with A/B testing to evaluate the recommendation performance. We also plan to improve the speed of model training, so that the model has better training efficiency when it is compatible with more features.
